# Prevalence and characteristics of foodborne pathogens from slaughtered pig carcasses in Korea

**DOI:** 10.3389/fvets.2023.1158196

**Published:** 2023-03-31

**Authors:** Serim Hong, Hye Jeong Kang, Hye-Young Lee, Hye-Ri Jung, Jin-San Moon, Soon-Seek Yoon, Ha-Young Kim, Young Ju Lee

**Affiliations:** ^1^College of Veterinary Medicine and Zoonoses Research Institute, Kyungpook National University, Daegu, Republic of Korea; ^2^Bacterial Disease Division, Animal and Plant Quarantine Agency, Gimcheon, Republic of Korea

**Keywords:** foodborne pathogen, pig, carcass, slaughterhouse, microbial quality

## Abstract

The introduction of bacteria into slaughterhouses can lead to microbial contamination in carcasses during slaughter, and the initial level of bacteria in carcasses is important because it directly affects spoilage and the shelf life. This study was conducted to investigate the microbiological quality, and the prevalence of foodborne pathogens in 200 carcasses from 20 pig slaughterhouses across Korea. Distribution of microbial counts were significantly higher for aerobic bacteria at 3.01–4.00 log_10_ CFU/cm^2^ (42.0%) and 2.01–3.00 log_10_ CFU/cm^2^ (28.5%), whereas most of *Escherichia coli* showed the counts under 1.00 log_10_ CFU/cm^2^ (87.0%) (*P* < 0.05). The most common pathogen isolated from 200 carcasses was *Staphylococcus aureus* (11.5%), followed by *Yersinia enterocolitica* (7.0%). In total, 17 *S. aureus* isolates from four slaughterhouses were divided into six pulsotypes and seven *spa* types, and showed the same or different types depending on the slaughterhouses. Interestingly, isolates from two slaughterhouses carried only *LukED* associated with the promotion of bacterial virulence, whereas, isolates from two other slaughterhouses carried one or more toxin genes associated with enterotoxins including *sen*. In total, 14 *Y. enterocolitica* isolates from six slaughterhouses were divided into nine pulsotypes, 13 isolates belonging to biotype 1A or 2 carried only *ystB*, whereas one isolate belonging to bio-serotype 4/O:3 carried both *ail* and *ystA*. This is the first study to investigate microbial quality and the prevalence of foodborne pathogens in carcasses from slaughterhouses nationally, and the findings support the need for ongoing slaughterhouse monitoring to improve the microbiological safety of pig carcasses.

## 1. Introduction

Foodborne illness is an important public health problem that causes an estimated 600 million illnesses and 420,000 deaths annually worldwide (1). In particular, food-producing animals are the major reservoirs of many foodborne pathogens such as Shiga toxin-producing *Escherichia coli, Salmonella* spp., *Staphylococcus aureus, Campylobacter* spp. *Yersinia* spp., and *Listeria monocytogenes* (2–4), and contamination of carcasses with foodborne pathogens can occur at several stages within the food production chain (5).

The slaughter stage has been a major focus of food safety interventions. Namely, the introduction of bacteria into slaughterhouses can lead to microbial contamination at several processing steps during slaughter. The initial opening of the carcass and the removal of highly contaminated organs, such as the intestines, pluck set and tonsils (6), increase the risk of microbial spread to carcass surfaces. Moreover, insufficient disinfection of cutting knifes or machinery can lead to cross-contamination between carcasses (7).

Recently, Bae et al. (8) reported the first study tracking foodborne pathogens in pigs and related pork products at all points along the pork supply chain, including farms, slaughterhouses, meat processing plants, and retail stores, in Korea. In particular, *Y. enterocolitica* and Shiga toxin-producing *E. coli* were the major pathogens isolated from carcasses in slaughterhouses. Moreover, Im et al. (9) reported that *S. aureus, Salmonella* spp., and *C. perfringens* were isolated from edible pig intestines in 11 pig slaughterhouses in Korea. According to the United States Department of Agriculture, evaluation of the hygiene status in pig slaughterhouses mainly targets *Salmonella* and other major pathogens (10). In Korea, foodborne pathogens surveillance at the slaughter stage has been conducted nationwide since 2010 to ensure food safety and reduce risks to human health. This study aimed to report on microbiological quality and distribution of foodborne pathogens in pig slaughterhouses nationwide and identify the genetic relationships and characteristics of major foodborne pathogens.

## 2. Materials and methods

### 2.1. Sample collection

In total, 200 carcasses were collected from 20 pig slaughterhouses across Korea between 2020 and 2021. In addition, pigs are produced mostly from three-way hybrids: Landrace, Yorkshire, and Duroc, and slaughtered at a live weight of ~110 kg. According to the Ministry of Food and Drug Safety (MFDS) protocol (11), a sterile sponge (Nasco, Fort Atkinson, WI, USA) hydrated with 10 ml of buffered peptone water (BPW; Difco, BD Biosciences, San Jose, CA, USA) was used to swab a 300 cm^2^ surface area composite that included one belly site (100 cm^2^), one ham site (100 cm^2^), and one jowls site (100 cm^2^) from each carcass cooled at 4°C for 24 h after slaughter. Ten carcasses were collected from each slaughterhouse, and all swab samples were transferred to the laboratory under 4°C conditions.

### 2.2. Bacterial count and isolation

Swab samples were inoculated in 30 ml of BPW and homogenized for 1 min using a stomacher (Stomacher 80 Biomaster, Seward, UK). To determine the aerobic bacteria and *E. coli* counts, aliquots containing serially diluted (10-fold) swab samples were performed using the TEMPO^®^ reader system (bioMérieux, Marcy l'Étoile, France), and Petrifilm plates (3M, St. Paul, MN, USA), respectively, according to the manufacturer's instructions. The isolation of foodborne pathogens was performed according to the standard microbiological protocol notified by the MFDS (11). Briefly, to isolate Shiga toxin-producing *E. coli, Campylobacter* spp., *S. aureus, C. perfringens*, and *Y. enterocolitica*, 1 ml of BPW was inoculated into each 9 ml of mEC with novobiocin (Merck, Darmstadt, Germany), Bolton broth (Oxoid, Basingstoke, UK) with laked horse blood (Oxoid), Tryptic soy broth (BD Biosciences) with 10% NaCl, Cooked meat medium (BD Biosciences), and Peptone sorbitol bile broth (Sigma-Aldrich, St. Louis, MO, USA), respectively, and incubated for 24 h at 37°C for *E. coli, S. aureus*, and *C. perfringens*; 48 h at 42°C for *Campylobacter* spp.; and 48 h at 30°C for *Y. enterocolitica*. For *Salmonella* spp., 10 mL of BPW was primarily incubated for 24 h at 37°C, and then 0.1 ml of pre-enriched BPW culture was inoculated in 10 ml of Rappaport–Vassiliadis broth (Oxoid) and incubated for 24 h at 42°C. For *L. monocytogenes*, 1 mL of BPW was also primarily inoculated in 9 ml of Listeria enrichment broth (BD Biosciences) and incubated for 24 h at 30°C, and then 0.1 ml of broth was secondarily enriched in 10 ml of Fraser broth (BD Biosciences) for 48 h at 37°C. All enriched media were streaked on Tellurite–Cefixime–Sorbitol MacConkey agar (Oxoid) for Shiga toxin-producing *E. coli*, Baird–Parker agar (Oxoid) supplemented with egg yolk tellurite emulsion (Oxoid) for *S. aureus*, Tryptose–Sulfite–Cycloserine agar supplemented with egg yolk emulsion (Oxoid) for *C. perfringens*, Cefsulodin–Irgasan–Novobiocin agar (BD Biosciences) for *Y. enterocolitica*, Xylose lysine tergitol−4 agar (BD Biosciences) for *Salmonella* spp., and Oxford agar (Oxoid) for *L. monocytogenes* followed by incubation for 24 h at 37°C. Modified campy blood–free agar (Oxoid) streaked for *Campylobacter* spp. was incubated for 48 h at 42°C. All suspect colonies were performed using polymerase chain reaction (PCR) with specific primers (Table 1) (12–16, 19, 20), and MALDI-TOF mass spectrometry (bioMérieux). If the same species isolates from the same origin showed the same antimicrobial susceptibility patterns, then only one isolate was randomly selected.

**Table 1:** Primer sequences used in this study.

**Bacteria**	**Target gene**	**Sequence (5'-3')**	**Size (bp)**	**Annealing (°C)**	**References**
*Campylobacter coli*	Random	F: AGGCAAGGGAGCCTTTAATC	364	54	(12)
		R: TATCCCTATCTACAAATTCGC			
*Campylobacter jejuni*	Random	F: CATCTTCCCTAGTCAAGCCT	773	54	(12)
		R: AAGATATGGCACTAGCAAGC			
*Clostridium perfringens*	*cpa*	F: GTTGATAGCGCAGGACATGTTAAG	402	55	(13)
		R: CATGTAGTCATCTGTTCCAGCATC			
*Listeria monocytogenes*	*Listeriolysin O*	F: GACATTCAAGTTGTGAA	560	55	(14)
		R: CGCCACACTTGAGATAT			
*Salmonella* spp.	*InvA*	F: TTTACGGTCTATTTTGATTTG	443	54	(15)
		R: TATGCTCCACAAGGTTAATG			
Shiga toxin-producing *Escherichia coli*	*stx1*	F: TTCGCTCTGCAATAGGTA	555	50	(16)
		R: TTCCCCAGTTCAATGTAAGAT			
	*stx2*	F: GTGCCTGTTACTGGGTTTTTCTTC	118	50	(16)
		R: AGGGGTCGATATCTCTGTCC			
*Staphylococcus aureus*	*clf A*	F: CTTGATCTCCAGCCATAATTGGTGG	638	55	(17)
		R: GCAAAATCCAGCACAACAGGAAACGA			
*Yersinia enterocolitica*	Y1-Y2	F: AATACCGCATAACGTCTTCG	330	62	(18)
		R: CTTCTTCTGCGAGTAACGTC			

### 2.3. Serotyping

*Salmonell*a spp. was serotyped using commercial *Salmonella* O, H-phase 1 and H-phase 2 antisera (Difco, Detroit, MI, USA) according to the Kauffmann–White scheme (17). *L. monocytogenes* was carried out using commercial antisera (Denka Seiken, Tokyo, Japan) against the serovars 1/2a, 1/2b, 1/2c, 3a, 3b, 3c, 4c, 4d/4e, and 4b/4e following the manufacturer's instructions. *Y. enterocolitica* was serotyped using commercial antisera polyvalent group O:1–2, O:3, O:5, O:8, and O:9 (Denka Seiken) following the manufacturer's instructions.

### 2.4. Antimicrobial susceptibility testing

The minimum inhibitory concentrations (MICs) of 17 and 14 antimicrobial agents for *S. aureus* and *Y. enterocolitica*, respectively, were determined by the broth microdilution method using the commercially available Sensititre^®^ panels EUST (TREK Diagnostic Systems, West Sussex, UK) and CMV3AGNF (TREK Diagnostic Systems), respectively, following the manufacturer's instructions. MICs were interpreted according to the Clinical and Laboratory Standards Institute guidelines M100 (18), and *Y. enterocolitica* followed breakpoints in *Enterobacteriaceae*. *S. aureus* ATCC 25923 and *E. coli* ATCC 25922 were used as quality-control strains for *S. aureus* and *Y. enterocolitica*, respectively.

### 2.5. Detection of toxin and virulence genes

PCR amplification was performed to detect toxin genes in *S. aureus* and virulence genes in *Y. enterocolitica* as described by Van Duijkeren et al. (21) and Platt-Samoraj et al. (22), respectively. The toxin genes included those encoding enterotoxins (*sea, seb, sec, sed, see, seg, seh, sei, sej, sek, sel, sem, sen, seo, sep, seq*, and *ser*), leukotoxin family (*lukED*), exfoliative toxins (*eta* and *etb*), toxic shock syndrome toxin (*tsst-1*), and panton–valentine leukocidin (*pvl*), and the virulence genes included attachment invasion locus (*ail*), *Yersinia* stable toxin A (*ystA*), and *ystB*.

### 2.6. *S. aureus* protein A typing and biotyping

*Staphylococcus aureus* protein A (*spa*) typing was performed using as described by Shopsin et al. (23) using Ridom StaphType (Ridom GmbH, Wurzburg, Germany; www.spaserver.ridom.de). The biotyping of *Y. enterocolitica* was performed using lipase, esculin, indole, xylose, trehalose, pyrazinamidase, and Voges–Proskauer biochemical tests according to methods described by Weagant et al. (24).

### 2.7. Pulsed-field gel electrophoresis

According to the Centers for Disease Control and Prevention PulseNet protocol (25), DNA was digested by *Sma*I (Takara Bio Inc., Shiga, Japan) for *S. aureus* and by *Asc*I (Thermo Fisher Scientific, Waltham, MA, USA) for *Y. enterocolitica*. Electrophoresis was performed using the CHEF-DRIII pulsed-field gel electrophoresis (PFGE) system (Bio-Rad Laboratories, Hercules, CA, USA), and PFGE banding profiles were analyzed using Bionumerics software version 8.0 (Applied Maths, Sint-Martens-Latem, Belgium). Relatedness was calculated using the unweighted pair-group method with arithmetic averages algorithm based on the Dice similarity index, and a similarity coefficient of 90% was fixed to assemble PFGE clusters.

### 2.8. Statistical analysis

Pearson's chi-square test with Bonferroni correction were performed using the Statistical Package for Social Sciences version 26 (IBM Corp., Armonk, NY, USA). Differences were considered significant at *P* < 0.05.

## 3. Results

### 3.1. Distribution of levels of aerobic bacteria and *E. coli*

The distribution of aerobic bacteria and *E. coli* counts in pig carcasses is presented in Table 2. Distribution of microbial counts were significantly higher for aerobic bacteria at 3.01–4.00 log_10_ CFU/cm^2^ (42.0%) and 2.01–3.00 log_10_ CFU/cm^2^ (28.5%), whereas most of *E. coli* showed the counts under 1.00 log_10_ CFU/cm^2^ (87.0%) (*P* < 0.05).

**Table 2:** Distribution of microbial counts in 200 carcasses from 20 pig slaughterhouses.

**Count interval (log_10_ CFU/cm^2^)**	**No. pig carcass (%)**	
	**Aerobic bacteria**	***E. coli***
≤ 1.00	0 (0)^C^	174 (87.0)^A^
1.01–2.00	6 (3.0)^B,C^	11 (5.5)^B^
2.01–3.00	57 (28.5)^A^	12 (6.0)^B^
3.01–4.00	84 (42.0)^A^	3 (1.5)^B,C^
4.01–5.00	16 (8.0)^B^	0 (0)^C^
5.01–6.00	17 (8.5)^B^	0 (0)^C^
6.01–7.00	14 (7.0)^B^	0 (0)^C^
≥ 7.01	6 (3.0)^B,C^	0 (0)^C^

Values with different superscript letters represent significant differences between columns (*P* < 0.05).

### 3.2. Prevalence of foodborne pathogens

The prevalence of foodborne pathogens in slaughterhouses and pig carcasses is presented in Table 3. The most common pathogen isolated from 200 carcasses was *S. aureus* (11.5%), followed by *Y. enterocolitica* (7.0%), *C. perfringens* (4.0%), and *C. coli* (4.0%), and the most prevalent pathogens in 20 slaughterhouses were *S. aureus* (40.0%), *Y. enterocolitica* (30.0%), and *C. perfringens* (25.0%) (*P* < 0.05). In particular, *Y. enterocolitica* was divided into three serotypes, and *Y. enterocolitica* O:5 showed the highest prevalence in slaughterhouses (25.0%) and carcasses (5.0%). Moreover, two *C. coli* and one *S*. Agona isolates were obtained from two (10.0%) and one (5.0%) slaughterhouses, respectively, and three *L. monocytogenes* isolates obtained from three slaughterhouses were divided into three serotypes: 1/2a, 1/2b, and 1/2c.

**Table 3:** Prevalence of foodborne pathogens in pig slaughterhouses and carcasses.

**Pathogen**	**No. positive samples (%)**	
	**Slaughterhouses (*****n*** = **20)**	**Carcasses (*****n*** = **200)**
*Campylobacter coli*	2 (10.0)^A,B^	8 (4.0)^A,B,C^
*Campylobacter jejuni*	0 (0)^B^	0 (0)^C^
*Clostridium perfringens*	5 (25.0)^A,B^	8 (4.0)^A,B,C^
*Listeria monocytogenes*	3 (15.0)^A,B^	3 (1.5)^B,C^
*Listeria monocytogenes* 1/2a	1 (5.0)	1 (0.5)
*Listeria monocytogenes* 1/2b	1 (5.0)	1 (0.5)
*Listeria monocytogenes* 1/2c	1 (5.0)	1 (0.5)
*Salmonella* Agona	1 (5.0)^A,B^	1 (0.5)^C^
*Staphylococcus aureus*	8 (40.0)^A^	23 (11.5)^A^
Shiga toxin-producing *Escherichia coli*	0 (0)^B^	0 (0)^C^
*Yersinia enterocolitica*	6 (30.0)^A,B^	14 (7.0)^A,B^
*Yersinia enterocolitica* O:3	1 (5.0)	1 (0.5)
*Yersinia enterocolitica* O:5	5 (25.0)	10 (5.0)
*Yersinia enterocolitica* O:untypable	2 (10.0)	3 (1.5)

Values with different superscript letters represent significant differences between columns (*P* < 0.05).

### 3.3. Characteristics of *S. aureus* and *Y. enterocolitica*

**Figure 1:**
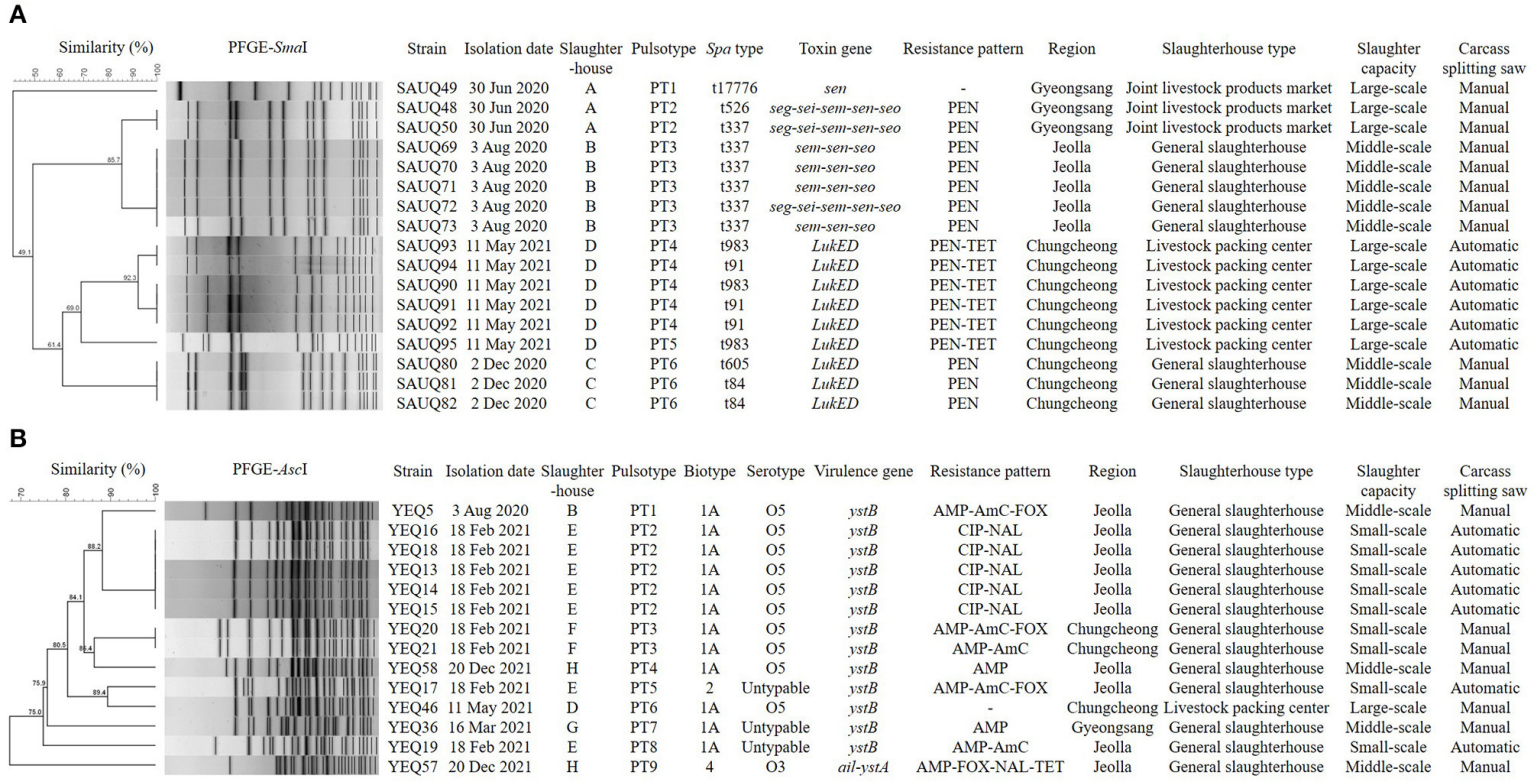
Dendrogram showing genetic relationships among the strains characterized by PFGE profiles **(A)** *Staphylococcus aureus*, **(B)** *Yersinia enterocolitica*. Showing similarities of < 90% in PFGE were considered to be unrelated. The types of slaughterhouses are divided into Livestock packing center (slaughterhouse for slaughter, processing, and sale), Joint livestock products market (slaughterhouse for slaughter and sale), and General slaughterhouse (slaughterhouse for slaughter). Slaughter capacities (pigs/day) are divided into small-scale (≤ 900), middle-scale (901–1,500), and large-scale (≥ 1,501). PEN, Penicillin; TET, Tetracycline; AMP, Ampicillin; AmC, Amoxicillin/ Clavulanic acid; FOX, Cefoxitin; CIP, Ciprofloxacin; NAL, Nalidixic acid.

Characteristics of phylogenetic, antibiotic resistance, and biotypic profiles of two major pathogens, *S. aureus* and *Y. enterocolitica* are presented in Figure 1. In total, 17 *S. aureus* isolates from four slaughterhouses were divided into six pulsotypes and seven *spa* types, and showed the same or different types depending on the slaughterhouses. Specifically, five isolates from slaughterhouse B showed the same pulsotype and *spa* type, but those from slaughterhouse D could be divided into two pulsotypes and two *spa* types. Furthermore, isolates from slaughterhouses A and C divided into two and one pulsotypes, and three and two *spa* types, respectively. Interestingly, isolates from slaughterhouses C and D only carried *LukED* associated with the promotion of bacterial virulence, whereas those from slaughterhouses A and B carried one or more toxin genes associated with enterotoxins, including *sen*. In total, 14 *Y. enterocolitica* isolates from six slaughterhouses were divided into nine pulsotypes, but isolates showed three biotypes and two serotypes, excluding three O:untypable isolates. Moreover, 13 isolates belonging to biotype 1A or 2 carried only *ystB* encoding an enterotoxin, and one isolate belonging to bio-serotype 4/O:3 carried both *ail* and *ystA*, which encode an attachment invasion locus and enterotoxin, respectively.

## 4. Discussion

Microbial contamination of meat is unavoidable because microorganisms are present on animals and in their environments. Thus, the initial level of bacteria in carcasses is important because as it directly affects spoilage and the shelf life (5). According to the Livestock Products Sanitary Control Act and Modernization of Swine Slaughter Inspection (10, 26), the hygienic quality of pig carcass is considered satisfactory when aerobic bacteria and *E. coli* counts are < 5.00 log_10_ CFU/cm^2^ and < 4.00 log_10_ CFU/cm^2^, respectively. In this study, although 163 (81.5%) among 200 carcasses met this criterion for aerobic bacterial counts, 37 (18.5%) carcasses showed aerobic bacterial counts exceeding 5.00 log_10_ CFU/cm^2^. Lebret and Candek-Potokar (27) reported that microbial growth in pork carcasses depends on the environmental conditions during the aging process, and microbes ultimately affect pork spoilage and quality deterioration. Therefore, it is important to control microbial growth during storage by enhancing hygiene. In contrast, all carcasses had *E. coli* counts of < 4.00 log_10_ CFU/cm^2^, with 87.0% of carcasses showing counts of ≤ 1.00 log_10_ CFU/cm^2^. Van Ba et al. (28) previously reported that the average counts of aerobic bacteria and *E. coli* in pig carcasses in Korea were satisfactory, and Lindblad et al. (29) and Bohaychuk et al. (30) also reported that pig carcasses from Sweden and Canada met the criteria for aerobic bacteria and *E. coli* counts, respectively. In developed countries, risk factors in slaughterhouses are strictly managed because a high initial microbial load, poor hygiene practices, or high temperatures (>15°C) in the slaughtering lines can affect the distribution of microorganisms (31), and the quality of carcasses can be improved through food safety/HACCP implementation, non-conformities control, site hygiene, and pest control (32).

Several studies have reported that the most common foodborne pathogens associated with pigs are *Campylobacter* spp., *Salmonella* spp., *S. aureus, L. monocytogenes*, and *Y. enterocolitica* (33–35). In this study, the prevalence of seven major foodborne pathogens was investigated, and five pathogens were isolated from carcasses with *S. aureus* being the most prevalent (40.0% of slaughterhouses and 11.5% of carcasses). The MFDS (36) reported 21 cases of staphylococcal food poisoning involving 378 patients in Korea from 2018 to 2022. *S. aureus* is one of the most common causes of food poisoning, and is commonly found on the skin and in the mucous membranes of human beings and animals, and is particularly dangerous at slaughterhouses because of its potential for transmission from animals to slaughter operators and *vice-versa* (37). In Germany, Greece, and South Africa, the prevalence of *S. aureus* in pig carcasses were reported to be 6.0%, 15.5%, and 32.5%, respectively (38–40). The high prevalence of *S. aureus* in pig carcasses may be related to a lack of skinning of pigs during slaughter; therefore, it is important to minimize skin contamination during slaughter.

In this study, the second most frequently isolated pathogen was *Y. enterocolitica* (30.0% of slaughterhouses and 7.0% of carcasses). In the United States, *Y. enterocolitica* is estimated to cause ~117,000 cases of illnesses, 640 hospitalizations, and 35 deaths each year (41). In Europe, human yersiniosis is the third most common foodborne zoonotic disease after campylobacteriosis and salmonellosis (42). Pigs are considered the primary reservoirs of human yersiniosis globally because pigs are the only animal species from which pathogenic strains have frequently been isolated so far (43). Moreover, pigs infected with *Y. enterocolitica* shed the organism in feces on farms for prolonged periods, and *Y. enterocolitica* has been frequently isolated from the tonsils of pigs at slaughter (44). Therefore, pig carcasses can be contaminated based on their infected tissues and intestinal contents in slaughterhouses (45).

*C. perfringens* is one of the bacterial hazards identified in the Guides to Good Hygiene Practices, and of application of HACCP principles in the slaughtering because *C. perfringens* frequently colonizers of the intestinal tracts of various food animals (46). In this study, the prevalence of *C. perfringens* was only 4.0% in carcasses, but it was detected in as high as 25.0% of slaughterhouses. Therefore, for hygienic carcass production, it is necessary to fast animals on the farm before slaughter and reduce the contents of the gastrointestinal during slaughter (47).

*Campylobacter jejuni* was not found in this study, but *C. coli* was identified in 4.0% of carcasses. Several researchers have reported that the predominant species of *Campylobacter* in pigs is *C. coli*, whereas that in poultry and cattle is *C. jejuni* (48, 49). *Campylobacter* spp. do not usually cause clinical signs in animals, but reducing the abundance of *Campylobacter* in carcasses at slaughter can be an important step in the farm-to-table continuum through which *Campylobacter* enters the food chain.

Mechesso et al. (50) reported that the prevalence of *S*. *agona* was 10.8% in domestic pig carcasses from 2016 to 2018 in Korea, but in this study, this serovar was only isolated from one carcass. *S*. Agona is an important cause of food poisoning, and infection in humans usually occurs *via* the consumption of contaminated meat and eggs (51). Recently, Trinetta et al. (52) reported that the prevalence of *S*. Agona was 24.5% at 11 feed mills in eight states representative of the main pig production areas within the United States. Although the risk of feed-borne salmonellosis is difficult to quantify, this report indicated that *Salmonella* can be transmitted *via* contaminated feed through the food chain to pig farms, slaughterhouses, and ultimately to humans. Therefore, risk assessment studies for *Salmonella*-contaminated feed should be continuously performed to identify potential hazards.

In this study, the genetic and phenotypic characteristics of *S. aureus* and *Y. enterocolitica*, the main pathogens isolated from pig carcasses, were investigated. The prevalence of *S. aureus* and *Y. enterocolitica* had no relationship with the season, region, slaughterhouse type, slaughter capacity, and type of carcass splitting even though carcasses were collected from slaughterhouses across Korea (data not shown). Moreover, some isolates from the same slaughterhouse clustered in the same pulsotype, but 17 *S. aureus* isolates were ultimately divided into six pulsotypes. For identifying the epidemiological relevance of food poisoning bacteria, PFGE is generally preferred over other typing methods such as multilocus sequence typing or *spa* typing because standardization of the PFGE protocol has established a nomenclature for local pulsotypes in many countries (53), and because it is a highly discriminatory and valuable technique for the typing of classification within species (54).

The *S. aureus* isolates were divided into seven *spa* types, showing more diverse *spa* types than pulsotypes within the same slaughterhouse. Shopsin et al. (23) reported that *spa* typing provides clonal groupings that PFGE techniques cannot identify individually; thus, analyzing *spa* types together provides high differentiation in describing PFGE subtyping. In particular, among the *spa* types, t34 and t337 have been reported to be predominant in pigs worldwide (55), and t337 was also frequently confirmed in this study, consistent with previous reports (56).

In this study, 14 *Y. enterocolitica* isolates were divided into nine pulsotypes, whereas only three biotypes (1A, 2, and 5) and two serotypes (O3 and O5) were identified, excluding the untypable serotype. In general, *Y. enterocolitica* is divided into the non-pathogenic biotype 1A, weakly pathogenic biotypes 2–5, and highly pathogenic biotype 1B (57). Fortunately, biotype 1B was not identified in this study. *Y. enterocolitica* O:5, which was the most common serogroup in this study, has been reported to be the most prevalent serogroup worldwide, and it is mainly associated with non-pathogenicity type (16, 44). However, *Y. enterocolitica* bio-serotype 4/O:3, which was isolated from only one carcass in this study, is known to be pathogenic to humans (58).

In this study, all *S. aureus* isolates carried staphylococcal enterotoxin genes that induce food poisoning or leukotoxin genes that promote virulence (59). These two genes are located on mobile elements in bacterial genomes such as plasmids or pathogenic islands; thus they can easily be transferred horizontally between strains (60, 61).

In *Y. enterocolitica*, pathogenicity is associated with the presence of plasmids and chromosomal virulence genes, but the most commonly encoded virulence determinants are *ail* and the enterotoxin-encoding gene y*st*, which are chromosomal virulence markers (62). In particular, *Y. enterocolitica* biotype 1A, which is regarded as a non-pathogenic environmental strain, lacks the pYV plasmid (plasmid of *Yersinia* virulence) and most chromosomal virulence markers (63). In this study, all 12 *Y. enterocolitica* biotype 1A isolates harbored only *ystB*, which is recovered from wild animals and the environments, as previously described (64). However the *ail*, which usually accompanies *ystA* in pathogenic *Y. enterocolitica*, was identified in one *Y. enterocolitica* bio-serotype 4/O:3 isolate.

In this study, all *S. aureus* except one isolate showed the resistance to penicillin, and isolates from slaughterhouse D showed the resistance to both penicillin and tetracycline. Moreover, *Y. enterocolitica* showed the resistance to various antimicrobial subclasses, although there were similarities in antimicrobial resistant classes by slaughterhouse. The difference in the resistance of isolates by slaughterhouse is presumed to be attributable to differences in the antimicrobial classes mainly used in farms by region, because pigs are mainly slaughtered at slaughterhouses located in the region in which they are raised.

This is the first study to investigate microbial quality and the prevalence of foodborne pathogens in carcasses from slaughterhouses nationally, and the findings support the need for ongoing slaughterhouse monitoring to improve the microbiological safety of pig carcasses.

## Data availability statement

The original contributions presented in the study are included in the article/supplementary material, further inquiries can be directed to the corresponding authors.

## Ethics statement

Ethical review and approval was not required for the study on animals in accordance with the local legislation and institutional requirements. Written informed consent from the owners for the participation of their animals in this study was not required in accordance with the national legislation and the institutional requirements.

## Author contributions

SH, J-SM, S-SY, H-YK, and YL conceived and designed all the experiments. SH, HK, H-YL, H-RJ, and H-YK participated in collecting the data and performing the tests. SH, H-YK, and YL analyzed the data and drafted the manuscript. All authors read and approved the final manuscript.
